# Dynamic reciprocal interactions between activated T cells and tumor associated macrophages drive macrophage reprogramming and proinflammatory T cell migration within prostate tumor models

**DOI:** 10.1038/s41598-024-75265-9

**Published:** 2024-10-16

**Authors:** Erika Heninger, Matthew Thomas Breneman, Emma Elizabeth Recchia, Sheena Catherine Kerr, Reyna Elvan Dogru, Marina Nasrin Sharifi, Aaron Matthew LeBeau, David Kosoff

**Affiliations:** 1 Carbone Cancer Center, University of Wisconsin-Madison, Madison, WI USA; 2 Department of Pathology and Laboratory Medicine, University of Wisconsin-Madison, Madison, WI USA; 3 Department of Medicine, University of Wisconsin-Madison, Madison, WI USA; 4 William S Middleton Memorial Veteran’s Hospital, Madison, WI USA; 5 Department of Medicine, Carbone Cancer Center, University of Wisconsin Madison, 1111 Highland Avenue, WIMR 7105, Madison, WI USA

**Keywords:** Tumor microenvironment, TAM, T cells, Macrophage, Co-culture, Molecular medicine, Cancer microenvironment, Tumour immunology, Translational research

## Abstract

Tumor-associated macrophages (TAMs) have been implicated as a tumor microenvironment (TME) cell population, which may be playing a vital role in the inhibition of effective T cell responses in the prostate TME. In this manuscript, we leverage a novel microscale cell culture platform, known as Stacks, to investigate mono-, co-, and tri-culture TME models comprised of prostate tumor cell lines, primary macrophages, and autologous T cells from patients with prostate cancer. Through multiplexed analysis of these multi-cellular prostate tumor models, we capture a dynamic interaction between primary TAMs and activated T cells that resulted in reciprocal proinflammatory activation of both cell populations upon interaction. These findings suggest that activated T cells are capable of reprogramming immunosuppressive TAMs in the context of prostate tumor models and that TAM reprogramming may play a key supportive role in restoring proinflammatory T cell tumor responses in the prostate TME.

**Supplementary Information:**

The online version contains supplementary material available at 10.1038/s41598-024-75265-9.

## Introduction

Therapies that enhance anti-tumor immune responses are a rapidly evolving class of systemic therapeutic agents that have a complex role in cancer treatment. For some cancers, immune therapies have been shown to induce dramatic and durable tumor regressions and are now considered standard of care treatments^1–3^. For other types of cancers, however, immunotherapies have a very limited role in treatment and are rarely used due to a lack of clinical benefit. Prostate cancer, which remains the 2nd leading cause of cancer-related death in men in the US, is one of the most notable examples of a cancer that has been largely unresponsive to these treatments. A variety of immune therapeutic strategies have been investigated for patients with prostate cancer both as monotherapies or in combination with chemotherapy, hormone therapies, or radiation therapy. However, none have demonstrated more than modest treatment response or survival benefit in large clinical trials^4–11^. The limited benefit of such a diverse array of immune therapeutic approaches suggests that prostate tumors utilize potent immunosuppressive pathways to subvert immune control and that these pathways cannot be overcome by conventional immune therapies alone.

Many of the immunotherapeutic strategies currently utilized in clinical practice depend on the effective infiltration of tumor-directed T cells within the tumor microenvironment (TME)^12^. Unfortunately, histologic evaluation of the prostate TME has demonstrated that even with the utilization of immunotherapies, T cells have a limited ability to migrate beyond the interface of tumor and surrounding tissue and there is limited immune cell infiltration within prostate tumor foci compared to normal adjacent tissue^13–15^. Furthermore, T cells that infiltrate prostate tumors have been found to be either immunosuppressive or limited in their function to exert anti-tumor response^16,17^. Investigation into the mechanisms that underlie immune dysfunction in prostate tumors has identified tumor-associated macrophages (TAMs) as a cell population with a complex and potent role in the regulation of T cell recruitment and function within the TME^13,18,19^. While in some cancers such as colorectal TAMs have been shown to express a pro-inflammatory, M1-like phenotype and are associated with better prognoses, in prostate cancer, TAMs have been associated with the predominantly immunosuppressive M2-like phenotype and worse clinical outcomes^20–23^. The predominance of immunosuppressive TAMs within the prostate TME suggests that these cells may play a key role in T cell inhibition in prostate cancer. However, due to the diversity of TAM phenotypes and the complexity of the prostate TME, prior research has been unable to clearly delineate the role of TAMs in prostate cancer immunosuppression.

In this study, our primary aim was to test our hypothesis that TAMs play a key role in inhibiting tumor-directed T cell responses in the prostate TME. To investigate this hypothesis, we leveraged a novel microscale platform, known as Stacks, which we have previously validated for high-throughput, multiplexed, spatiotemporal analysis of multi-cellular TMEs^24–27^. Through the utilization of the Stacks platform, we developed mono-, co-, and tri-culture model configurations comprised of androgen-dependent or androgen-independent prostate cell lines with primary, autologous macrophages and T cells from patients with prostate cancer. These models included parallel analysis of up to 15 simultaneous culture conditions. Each of these models was evaluated through multi-analyte investigation, including mRNA expression, surface protein expression, secreted factors, and migration, representing a first-in-kind integrated in vitro analysis of the prostate tumor immune microenvironment. Our multi-analyte investigation captured a dynamic interaction between primary TAMs and activated T cells in multi-cellular prostate tumor models that resulted in reciprocal proinflammatory activation. These findings suggest that even in the context of prostate cancer, TAM reprogramming is possible and that re-polarizing TAMs may play a key supportive role in restoring proinflammatory T cell tumor responses in a feed-back mechanism.

## Results

### Primary TAMs increase tumor-directed T cell migration in androgen-dependent and independent prostate tumor models

**Fig. 1:**
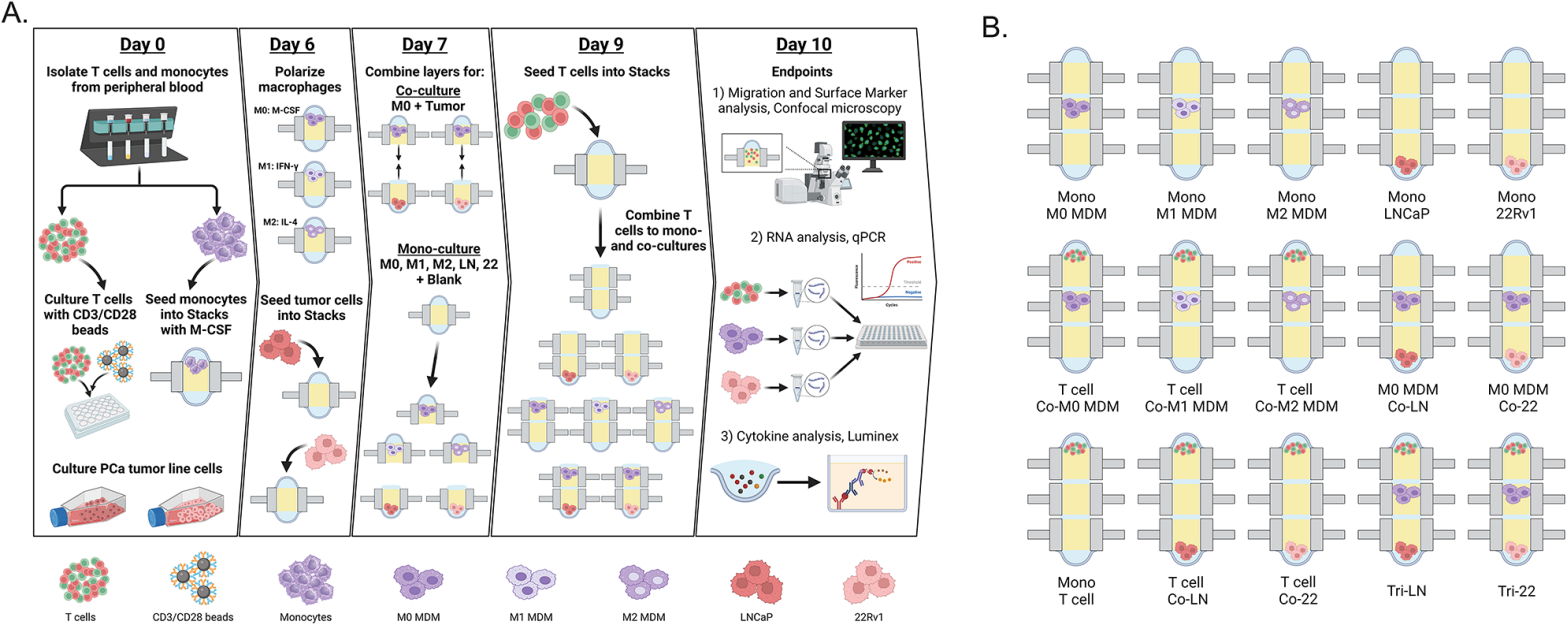
Stacks multi-culture workflow schematics. (**A**) Experimental workflow using three-layer Stacks platform including primary patient-derived T cells, monocyte-derived macrophages and tumor cell line cultures for multiple endpoint analyses. (**B**) Standard three-layer Stacks configurations to leverage multi-cellular co- and tri-culture combinations and spatial orientations.

To evaluate the effect of TAMs on T cell migration and phenotype in a multicellular prostate tumor model, we established mono-, co-, and tri-culture configurations that included tumor cells, macrophages, and T cells in the Stacks microfluidic system (Fig. 1A). Macrophages and T cells were primary autologous human cells derived from the peripheral blood from 25 donors with prostate cancer. 21 out of 25 donors had metastatic disease and 10 out of 25 patients had a castrate resistant status (Supplementary Table 1). The macrophages were derived from CD14^+^ monocytes and are hereafter referred to as monocyte-derived macrophages (MDMs). To simulate an activated T cell phenotype in our TME model, T cells were activated with anti-CD3/anti-CD28 (CD3/CD28) prior to culture in Stacks wells. Tumor cells included androgen-independent 22Rv1 and androgen-dependent LNCaP cell lines. For the tri-culture models, tumor cells and MDMs were co-cultured for 48 h to induce a TAM-like phenotype in the MDMs. Activated T cells were then added to a separate Stacks layer for 24 h followed by isolation of all layers for subsequent multi-analyte investigation that included T cell migration, mRNA analysis of distinct cell subsets and secreted factor profiling from cell culture supernatant. The standard configurations of mono-, co-, and tri-culture models are shown in a schematic figure (Fig. 1B). Briefly, in a 3-Stacks configuration, tumor cells were consistently seeded in the bottom layer, MDMs were seeded in the middle and T cells were added on the top layer. Cells for each individual layer were seeded on top of polymerized hydrogel in a 10 µl media droplet. By observation, most cell types embed the hydrogel within hours and the top droplet becomes cell-free. In mono-or co-cultures, empty wells contained hydrogel with media only to maintain standardized culture conditions through the same number of stacked layers within each configuration. Individual cell types seeded in separate Stacks layers were subsequently processed as individual culture components of our multi-cellular system by detachment of the stacked layers for analyses. Fluorescent microscopy steps including staining, washing and imaging were performed directly ‘in-chip’ on each individual detached layer without the removal of the cells from the culture wells. RT-qPCR analysis was performed on each individual cell subset by detaching the stacked layers followed by collagenase digestion of the hydrogel matrix and isolation of mRNA from each individual cell type from each individual well to represent specific cell subsets in our multi-cultures.

**Fig. 2:**
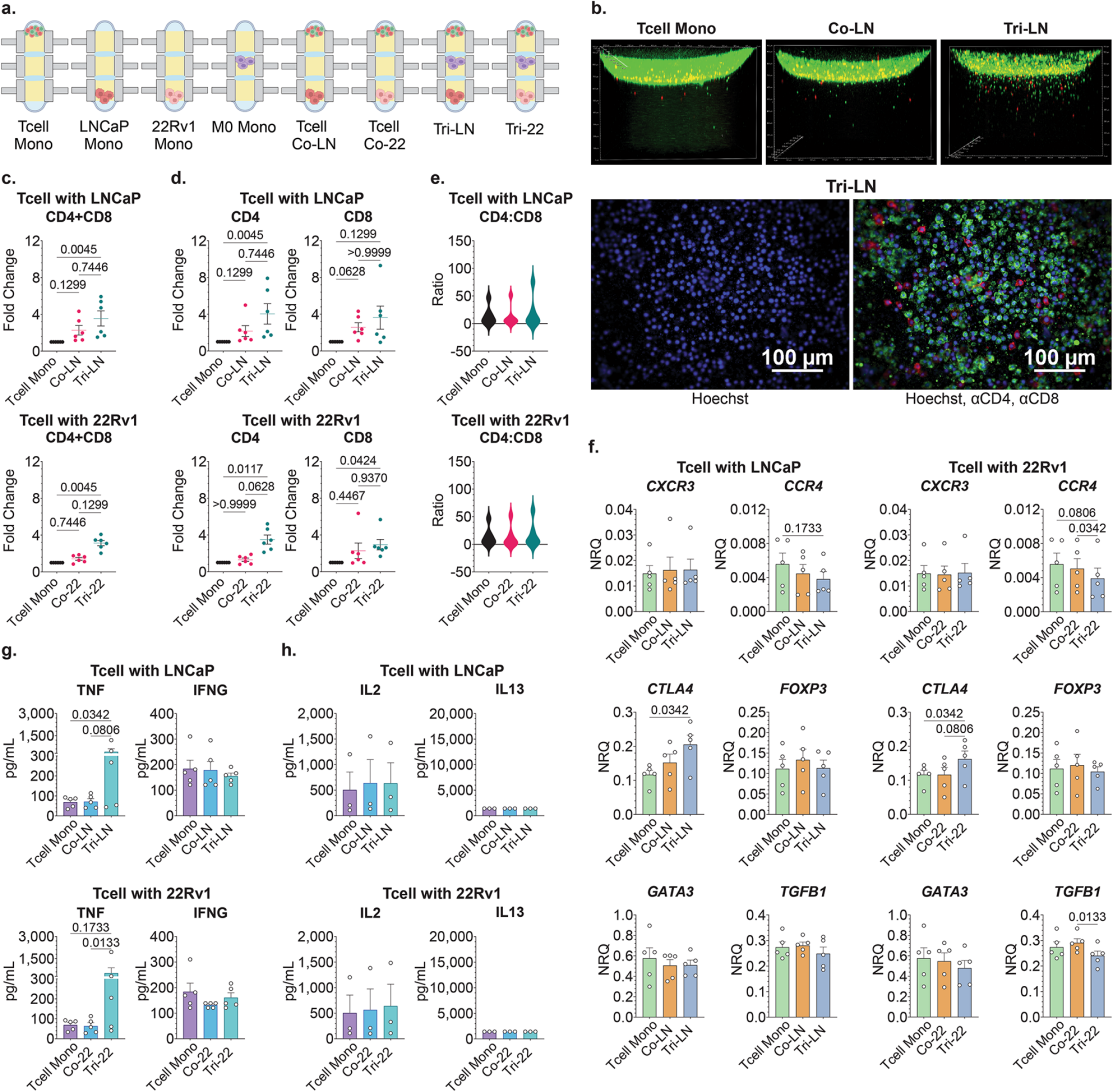
Analysis of T cells in multicellular TME model system. (**A**) Primary activated T cells were cultured in mono-culture (Mono/T cell Mono), or co-culture with established LNCaPs (Co-LN) or 22Rv1 (Co-22) tumor cells, or in tri-culture with established autologous M0 MDM cultures and tumor cells (Tri-LN or Tri-22) for 24 h. Additionally, M0 MDM were also cultured in mono-culture (M0 Mono) for 10 days, and tumor cells were mono-cultured for 4 days (LN Mono or 22 Mono, respectively). (B-E) T cell migration analysis was performed by confocal microscopy (**B**) condensed z-layers represent T cells stained with DNA Hoechst (blue), CD4 BB515 (green), and CD8 PE (red); (C-E) quantification of images with spot detection. Data represents fold change in T cell migration distance projected over matched T cell mono-culture condition for (**C**) total T cell population and (**D**) CD4 or CD8 T cell subsets, individually. (**E**) Violin plots show ratio of CD4 to CD8 absolute migration distances; (**C**–**E**) *n* = 6; Data represent mean ± SEM. (**F**) mRNA expression in T cells was interrogated in T cell mono-, tumor co-culture, and tumor-MDM tri-culture conditions. Data expressed as normalized relative quantity (NRQ) as related to house-keeping genes *RPLP0* and *POLR2A*; *n* = 5. (**G**,**H**) Cell culture supernatant was isolated from five unique cellular conditions (T cell Mono and Co- and Tri-cultures for both tumor models) and analyzed for secreted protein expression by multi-analyte bead assay. Data expressed as protein concentration (pg/mL). (**G**) *n* = 5, (**H**) *n* = 3; One-way ANOVA nonparametric with Friedman post-hoc analysis. Data represent mean ± SEM.

In T cell migration assays, we quantified the number of T cells trafficking in the hydrogel in mono-culture, in co-culture with tumor cells, and in tri-culture with tumor cells and autologous unpolarized M0 MDMs (Fig. 2A). The number of migrating T cells was enumerated using spot-detection of Hoechst^+^ nucleated events beyond the 400 μm threshold set from the top of the well, which corresponded to the average depth of curvature of the hydrogel surface. We first evaluated the impact of tumor cells on total T cell migration and found no evidence of tumor cells independently inhibiting T cell trafficking in our model systems (Fig. 2B,C). The addition of MDMs did not significantly change T cell migration over co-culture conditions, and we found no evidence of MDM-derived suppression of tumor-directed T cell migration. On the contrary, in the context of tumor cells, MDMs further enhanced a positive trend towards increased tumor-directed T cell trafficking when compared to T cell mono-cultures. This behavior was similar in both tumor models, 3.55 mean fold-change in Tri-LN over Mono, *p* = 0.045; 3.13 mean fold-change in Tri-22 over Mono, *p* = 0.045, respectively (Fig. 2C). Next, we assessed if the migratory behavior of CD4 or CD8 T cells was any different and found that increase in total T cell migration in the presence of the MDMs and tumor cells was due to a comparable increase in both subsets with a 3 to 4-times fold-over change in Tri-culture over matched monocultures in the context of both tumor cell lines (Fig. 2D,E). Overall, our data indicated that the MDMs did not attenuate the migration of activated T cells towards tumor cells. Rather, the addition of MDMs to our multi-cellular models synergized toward enhanced tumor-directed CD4 and CD8 T cell trafficking, signifying a supportive role for TAMs in augmenting activated T cell homing toward tumor targets.

### Primary TAMs promote pro-inflammatory skewing of autologous T cells in androgen dependent and independent prostate tumor models

T cells represent a diverse cell population with a potential to express pro- or anti-inflammatory phenotypes in the context of the TME. To investigate if the presence of TAMs in our models promoted a pro-inflammatory or immunosuppressive T cell profile, we performed multiplexed analysis of T cell mRNA expression and secreted chemokine/cytokine signatures. CD3/CD28 stimulated T cells express a highly activated pro-inflammatory profile^28^, therefore, our analysis focused on how the varying culture conditions impacted this phenotype and activation state. To assess this, we first measured T cell mRNA expression of various chemokine receptors that associate with anti-tumor or tumor-promoting immune responses. We found that expression of *CXCR3 (*C-X-C motif chemokine receptor 3), a homing receptor that is highly expressed on activated T cells to enable trafficking to inflammatory sites, remained high in our model irrespective of the presence or absence of tumor cells or autologous MDMs (Fig. 2F). However, when MDMs were added to co-cultures, we captured a significant upregulation in *CTLA*4 (cytotoxic T-lymphocyte associated protein 4), which was consistent with a response to inflammatory stimuli^29^. Next, we looked at markers that have been previously associated with anti-inflammatory or regulatory T cell function in the context of the prostate TME. Addition of MDMs to 22Rv1 tumor-T cell co-cultures resulted in a decrease in *CCR4 (*C-C motif chemokine receptor 4) expression (Co-22 vs. Tri-22, 0.0056 vs. 0.0039, *p* = 0.0342) with a similar trend observed in LNCaP tri-cultures. We also found a similar pattern in downregulation of *TGFB1 (*transforming growth factor beta 1) expression in the 22Rv1 Tri-cultures (Mono vs. Tri-22, 0.29 vs. 0.24, *p* = 0.0133). However, we observed no differences in *FOXP3* (forkhead box P3) expression in T cells in response to tumor cells or MDMs. Additional mRNA markers of T cell activation, phenotype and function including MLKL, CASP3, EOMES, EZH2, HAVCR2, KLRG1, PDCD1, TCF7 and TOX also remained largely unchanged in co- and triculture conditions (Supplementary Fig. 1).

To further investigate TME interactions in our multi-cellular configurations, we assessed secretory protein profiles in cell culture supernatants. We found a robust increase in tumor necrosis factor-alpha (TNF) secretion when MDMs were added to tumor–T cell co-cultures in the context of both 22Rv1 (Co-22 vs. Tri-22, 64 vs. 515 pg/ml, *p* = 0.0133, respectively) and LNCaP tumor cells (Mono vs. Co-LN, 70 vs. 404 pg/ml *p* = 0.0342, Co-LN vs. Tri-LN, 71 vs. 404 pg/ml, *p* = 0.08, respectively), the source of which could potentially be both T cells and MDMs. While we found no change in interferon-gamma (IFNG) secretion in any of the co- or tri-culture conditions (Fig. 2G), high levels of IFNG were maintained across all TME configurations. Additionally, no significant change in interleukin 2 (IL2), interleukin 4 (IL4), interleukin 13 (IL13), or interleukin 10 (IL10) cytokine secretion was observed (Fig. 2H and Supplementary Fig. 2). Supplementary material is to illustrate that the increased IL4 and IL10 expression captured in tri-culture compared to T cell mono-culture condition was likely due to elevated baseline expression of these cytokines by MDM cell populations and not increased secretion by T cells. Overall, the addition of MDMs to T cell-tumor cell co-cultures induced proinflammatory factors such as TNF secretion, elevated *CTLA4* mRNA expression which is known to increase in response to T cell activation and simultaneously decreased anti-inflammatory *CCR4* and *TGFB1* mRNA expression. These findings suggested that in addition to promoting the tumor-directed migration of activated T cells, MDMs also had the capability to enhance T cell activation and drive proinflammatory skewing in these TME tri-culture models.

### Activated autologous T cells can repolarize prostate TAMs to increase secretion of T cell recruitment factors within prostate TME models

**Fig. 3:**
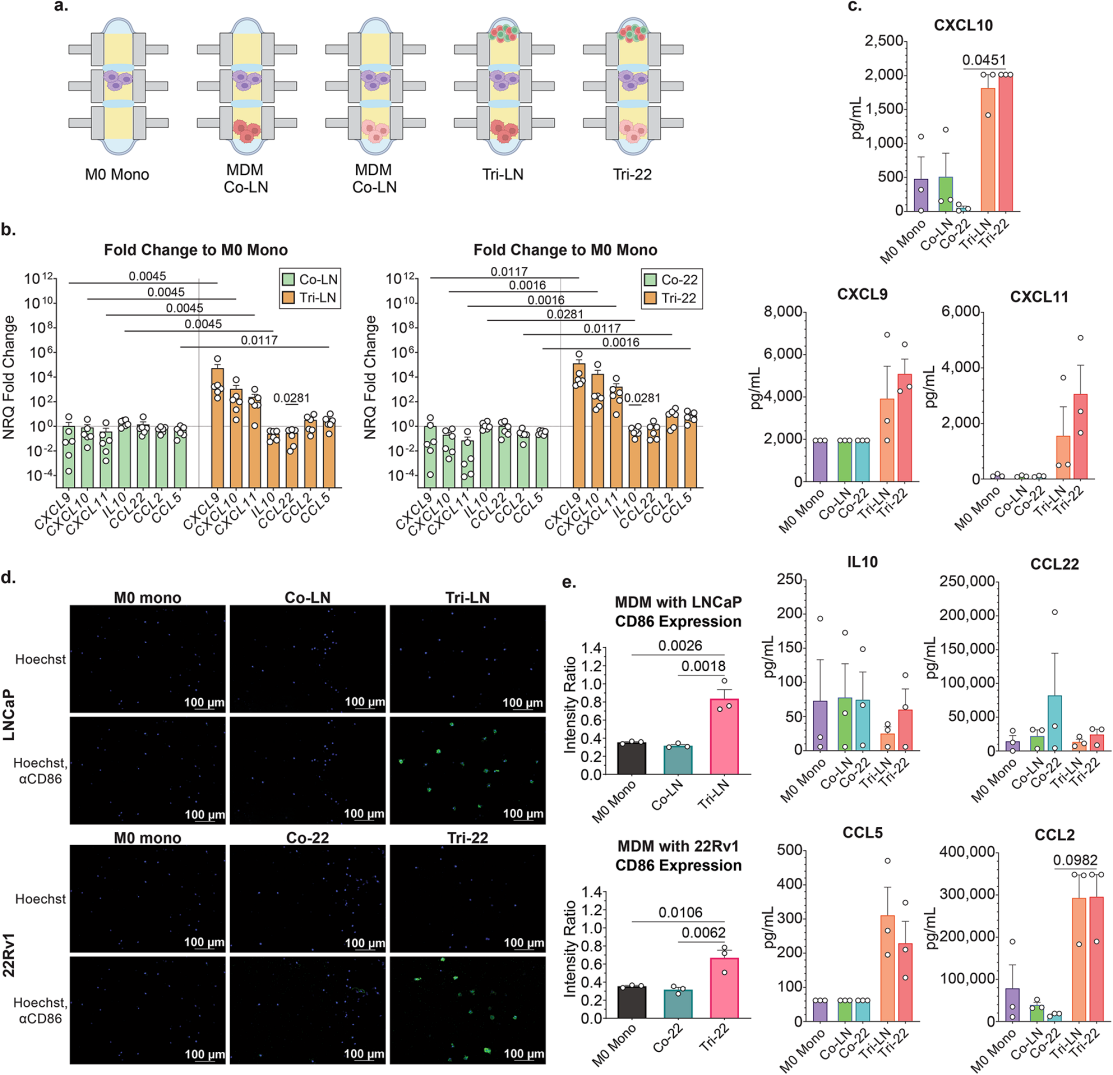
MDM analysis in multicellular TME model system. (**A**) Primary M0 MDMs were cultured in mono-culture MDM mono (M0) for 10 days, co-culture with established LNCaPs (Co-LN) or 22Rv1 (Co-22) cultures for 3 days, and tri-culture with autologous T cell cultures and established tumor cells (Tri-LN or Tri-22) for 24 h. (**B**) mRNA expression in MDMs was interrogated in MDM mono-, tumor co-culture, and tumor-MDM tri-culture conditions. Data represents fold change of NRQ expression as related to house-keeping genes *RPLP0* and *POLR2A *over matched MDM mono-culture levels (dashed line). Statistical analysis performed by One-way ANOVA nonparametric with Friedman post-hoc analysis *n* = 6. (**C**) Data represent secreted protein levels (pg/mL) using a multi-analyte bead assay. Statistical analysis was performed using One-way ANOVA nonparametric with Friedman post-hoc analysis comparing the mean rank of each column with the rank of every other column; *n* = 3; (**D**) CD86 surface protein expression was measured by ‘in-chip’ immunofluorescent staining and subsequent confocal microscopy in microdevice wells; CD86 Brilliant Blue 515 (BB5151; green) and Hoechst (blue). Representative cross-section images with (bottom row) or without (top row) anti-CD86 antibody channel shown as two-channel overlay. (**E**) Mean Fluorescent Intensity Ratio of CD86 binding of CD86^+^ cells projected to the nuclear Hoechst fluorescence mean intensity of all cells, for entire microwells in XYZ for each condition, in replicate. Cells were identified using NIS-Elements spot detection. Ordinary one-way ANOVA with post-hoc analysis comparing the mean rank of each column with the rank of every other column; *n* = 3; Data represent mean ± SEM.

To better understand the drivers behind TAM-derived induction of T cell migration and proinflammatory skewing captured in our model systems, we aimed to characterize the phenotype and function of the MDMs/TAMs in the various TME configurations. In order to investigate this, we analyzed MDM profiles in unpolarized baseline M0 MDM Mono-cultures, MDM-tumor cell Co-Cultures and MDM-tumor-T cell Tri-cultures (Fig. 3A). Analysis of mRNA and protein expression signatures demonstrated that in the presence of 22Rv1 cells, MDMs reduced expression of pro-inflammatory and T cell recruitment factors, including *CXCL10*, *CXCL11*, *CCL2* (C-C motif chemokine ligand 2), and *CCL5 (*C-C motif chemokine ligand 5), which was consistent with a TAM-like phenotype. Although similar patterns were observed in the LNCaP cultures, those were not statistically different. The addition of activated autologous T cells to tumor-MDM co-cultures resulted in a robust reversal of the baseline MDM/TAM phenotype with significantly increased expression of proinflammatory factors including *CXCL9* (C-X-C motif chemokine ligand 9), *CXL10* (C-X-C motif chemokine ligand 10), *CXCL11* (C-X-C motif chemokine ligand 11), and *CCL5* and reduction of *IL10* in tri-cultures over co-cultures in the context of both LNCaP and 22Rv1 tumor cells (Fig. 3B). Induction of MDM-derived *CCL2* mRNA expression was also detected in 22rv1 tri-cultures.

Next, we investigated if the change captured in MDM-derived T cell recruitment factors was reflected in secreted protein profiles (Fig. 3C). Although we found similar patterns in increasing CXCL9, CXCL10, CXCL11, CCL2 and CCL5 secretion in tri-cultures, these remained only trends likely due to inter-patient variability, therefore, future investigation is needed to gain a more conclusive picture of the MDM secretome in the context of activated T cells in the prostate TME.

To further assess MDM/TAM profiles, we evaluated expression of positive co-stimulatory molecule CD86, that is associated with proinflammatory immune responses and enhancement of anti-tumor T cell function^30^. Using confocal microscopy, we performed in-chip analysis of surface anti-CD86 binding of MDMs. Consistent with our findings of cytokine/chemokine profiles, the addition of activated T cells significant increased CD86 expression over matched mono-culture and co-culture conditions in the context of both LNCaP (intensity ratio Mono vs. Tri-LN, 0.35 vs. 0.84, *p* = 0.0026; Co-LN vs. Tri-LN, 0.32 vs. 0.84, *p* = 0.018, respectively) and 22Rv1 cells (Mono vs. Tri-22, 0.35 vs. 0.67, *p* = 0.0106; Co-LN vs. Tri-LN, 0.32 vs. 0.67, *p* = 0.0062, respectively) (Fig. 3D,E). This data, along with the noted changes in cytokine/chemokine expression, suggests that activated T cells can repolarize immunosuppressive TAMs towards a more pro-inflammatory phenotype in the context of a multicellular prostate TME.

**Fig. 4:**
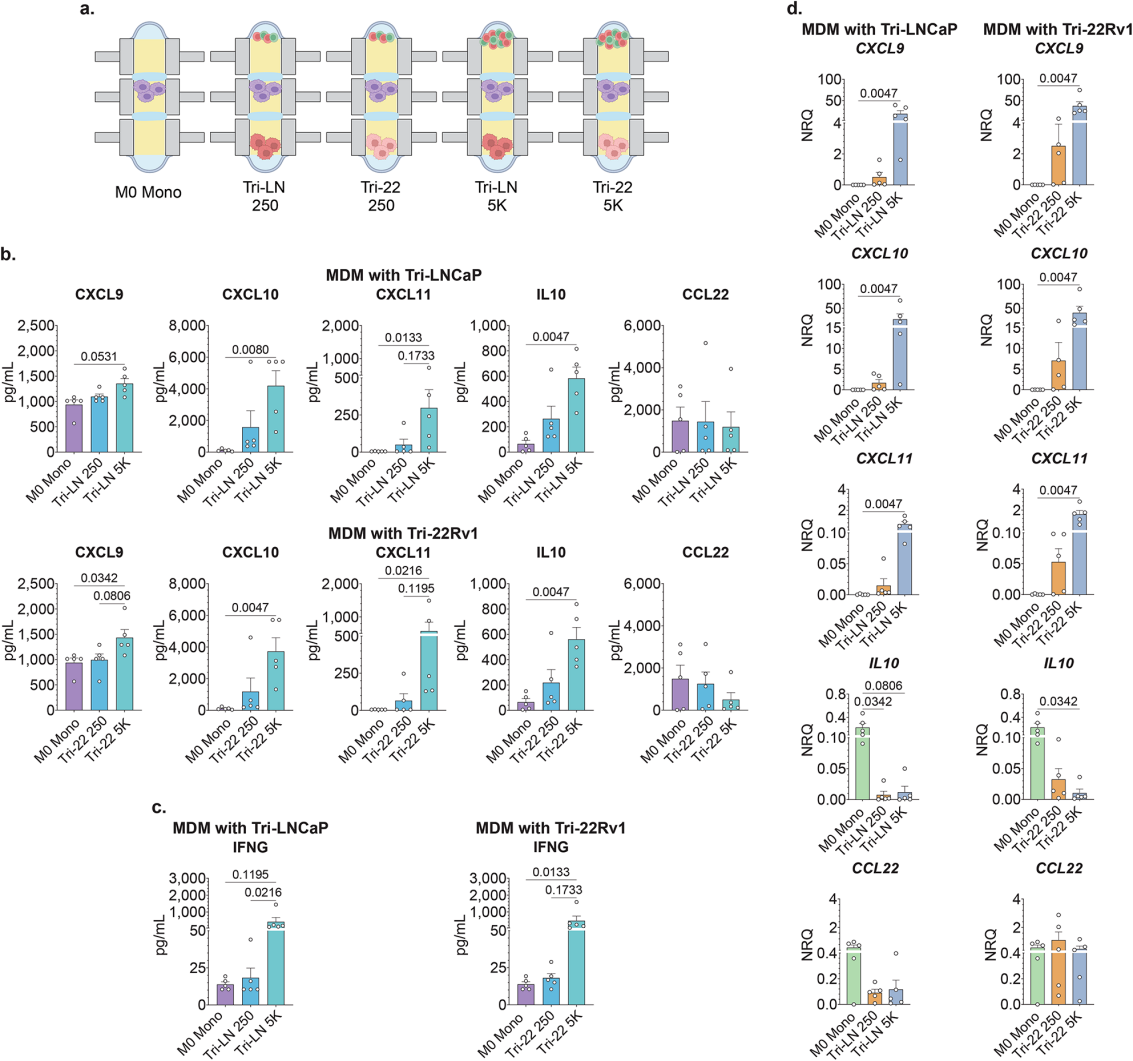
T cells affect MDM behavior. (**A**) Primary M0 MDMs were cultured either in mono-culture (M0 Mono) for 10 days; or cultured step-wise as mono-culture, to co-culture with established LNCaP or 22Rv1 tumor cells, followed by a 24 h tri-culture with autologous T cells at either 250 or 5000 cells per well (Tri-LN 250, Tri-22 250, Tri-LN 5 K, Tri-22 5 K). The M0 Mono culture condition served as shared, matched internal control for both tumor cell co-culture configurations, represented in both data sets. (**B**) mRNA expression in MDMs in mono-culture, and all four tri-culture conditions expressed as normalized relative quantity (NRQ), as related to house-keeping genes *RPLP0* and *POLR2A*. One-way ANOVA nonparametric with Friedman post-hoc analysis comparing the mean rank of each column with the rank of every other column; *n* = 5; error bar is mean with SEM. (**C**,**D**) Cytokine and chemokine protein concentrations (pg/mL) in cell cultures supernatant captured by multi-analyte bead assay. One-way ANOVA nonparametric with Friedman post-hoc analysis comparing the mean rank of each column with the rank of every other column; *n* = 5; Data represent mean ± SEM.

Since our tri-culture models simulated a rather robust activated T cell infiltrate, we next evaluated whether the T cell-mediated induction of the MDMs was limited by the number of the activated T cells present in the TME network. To accomplish this, we leveraged the low-input capabilities of Stacks to evaluate MDM phenotype with markedly reduced numbers of input activated T cells in the tri-culture configurations. For these assays, we compared the phenotype of the MDMs in unpolarized M0 monoculture to MDMs in tri-culture with LNCaP or 22Rv1 tumor cells and the presence of 250 or 5000 T cells per well (Fig. 4A). Analysis of MDM mRNA expression demonstrated that the addition of as low as 250 activated T cells to the tumor-MDM co-cultures induced an apparent positive trend in some proinflammatory cytokine/chemokine expression, and a clear negative trend in anti-inflammatory factors in MDMs (Fig. 4B). Remarkably, adding only 250 T cells to the Tri-cultures reduced MDM-derived *IL-10* mRNA expression in our LNCaP model (Mono MDM vs. 250 Tri-LN, NRQ 0.23 vs. 0.008, *p* = 0.0342, respectively). Although the degree of induction of most of the cytokines/chemokines only reached statistical significance in the context of 5000 T cells (Fig. 4B–D), some tendencies observed in the 250 T cell configuration suggested that even very low-level activated T cell presence could potentially re-set the TME to create an MDM polarization state. Further investigation is needed to determine the minimum threshold of activated T cells and/or T cell to MDM ratio present in the TME networks to produce enough drivers such as IFNG to establish a significant differential between Mono-MDMs and Tri-culture conditions.

### Neutralization of IFNG abrogates activated T cell reprogramming of TAMs

**Fig. 5:**
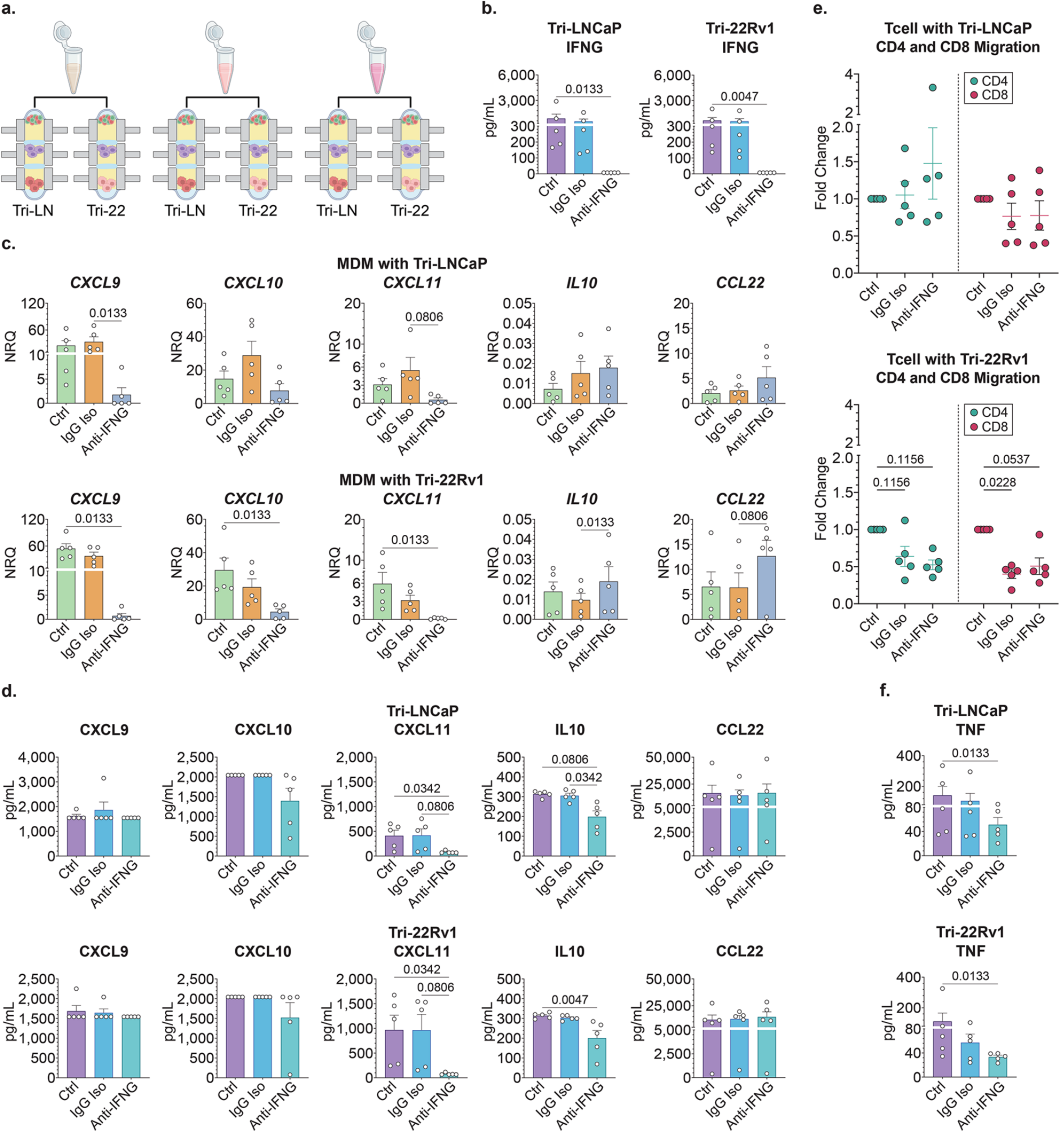
IFNG neutralization impacts T cell effect on MDMs. (**A**) Primary M0 MDMs were cultured stepwise in mono-culture, to co-culture with established LNCaP or 22Rv1 tumor cultures; T cells were added for the last 24 h with vehicle control (Ctrl), or IgG control (IgG Iso), or anti-IFNG neutralizing antibody (anti-IFNG). (**B**) IFNG protein concentration (pg/ml) in cell culture supernatants; *n* = 5; data expressed as mean ± SEM. (**C**) MDM mRNA expression in the six tri-culture cellular and treatment conditions shown as normalized relative quantity (NRQ) as related to house-keeping genes *RPLP0* and *POLR2A*. One-way ANOVA nonparametric with Friedman post-hoc analysis comparing the mean rank of each column with the rank of every other column; *n* = 5; Data represent mean ± SEM. (**D**,**F**) Concentrations of secreted cytokines and chemokines in supernatant from the six tri-culture cellular and treatment conditions, expressed as pg/mL. One-way ANOVA nonparametric with Friedman post-hoc analysis comparing the mean rank of each column with the rank of every other column; *n* = 5; Data represent mean ± SEM. (**E**) T cell migration analysis measured by in-chip confocal microscopy imaging in individual microdevice wells. Data is expressed as each sample’s fold change in migration distance as relative to its corresponding tri-culture vehicle control condition quantified by NIS-Elements. One-way ANOVA nonparametric with Friedman post-hoc analysis comparing the mean ranks of vehicle control IgG control, and vehicle control to Anti-IFNG neutralizing antibody; *n* = 5; Data represent mean ± SEM.

In our experiments, T cell derived changes in MDM/TAM profiles associated with an induction of proinflammatory chemokines such as CXCL9, CXCL10 and CXCL11. IFNG has been previously identified as a strong inducer of these chemokines and TNF has been shown to synergize with that effect although induced the expression of these cytokines at a lower magnitude as a single agent^31,32^. We have captured high levels of IFNG secretion in Tri-cultures, therefore, we hypothesized that T-cell derived IFNG was one of the main drivers of changes in MDM/TAM polarization in our prostate TME model system. To test this hypothesis, we investigated whether IFNG neutralization could abrogate MDM reprogramming in the triculture configurations. Tumor cells and M0 MDMs were co-cultured for 48 h prior to the addition of activated autologous T cells resuspended in media containing IFNG neutralizing antibody or IgG isotype control antibody, or PBS vehicle (Fig. 5A). Cells were then co-cultured for an additional 24 h prior to separation of Stacks layers for analysis.

First, we assessed the efficiency of IFNG neutralization in our model and found that anti-IFNG treatment reduced detectable protein levels significantly, consistent with the expected biological effect (Fig. 5B). Analysis of the MDMs demonstrated that IFNG neutralization diminished induction of *CXCL9*, *CXCL10* and *CXCL11* gene expression in MDMs in both tumor model systems. However, the level of reduction was variable between genes and cell lines with stronger effects captured in the 22Rv1 models. There was no significant difference in *IL10* or *CCL22* mRNA expression compared to control conditions aside from an increase in *IL10* mRNA expression compared to the isotype control in the 22Rv1 model (IgG iso vs. anti-IFNG, 0.0096 vs. 0.189, respectively; *p* = 0.0133) (Fig. 5C). Following administration of the neutralizing antibodies, we detected a reduction in CXCL11 secretion compared to vehicle control (Fig. 5D). We also found that IFNG neutralization abrogated the increase in IL10 secretion, suggesting that *IL10* induction was likely a compensatory response to the upregulation of proinflammatory pathways. Although both negative controls including vehicle and IgG isotype behaved similarly across most molecular analyses, it is important to note that our findings were somewhat limited by more variability captured in the isotype IgG control groups. Therefore, further investigation is needed to solidify our findings that will require more data points and/or assessing alternative isotype control IgG for neutralization experiments. Overall, our results supported our original hypothesis that TAM reprogramming in the presence of activated T cells was at least partly due to T cell-derived IFNG secretion.

Next, we assessed if IFNG neutralization had any effect on TAM-mediated CD4 and CD8 T cell migration, however, our findings were largely inconclusive part due to data variability and to similar effects captured in the isotype control and neutralizing antibody conditions. Therefore, the effect of IFNG neutralization on T cell migration requires further investigation (Fig. 5E). However, we did find that IFNG neutralization associated with a significant decrease in TNF secretion compared to the vehicle treated triculture control (Fig. 5F). This suggested that the diminished reprogramming of the MDMs also led to attenuation in other elements of the proinflammatory polarization feedback loop from T cells. Although TNF neutralization was not studied in our model, future studies should assess the synergy between IFNG and TNF behind pro-inflammatory MDM reprogramming.

### Activated autologous T cells repolarize M2-like MDMs to increase secretion of T cell recruitment factors within prostate tumor models

**Fig. 6:**
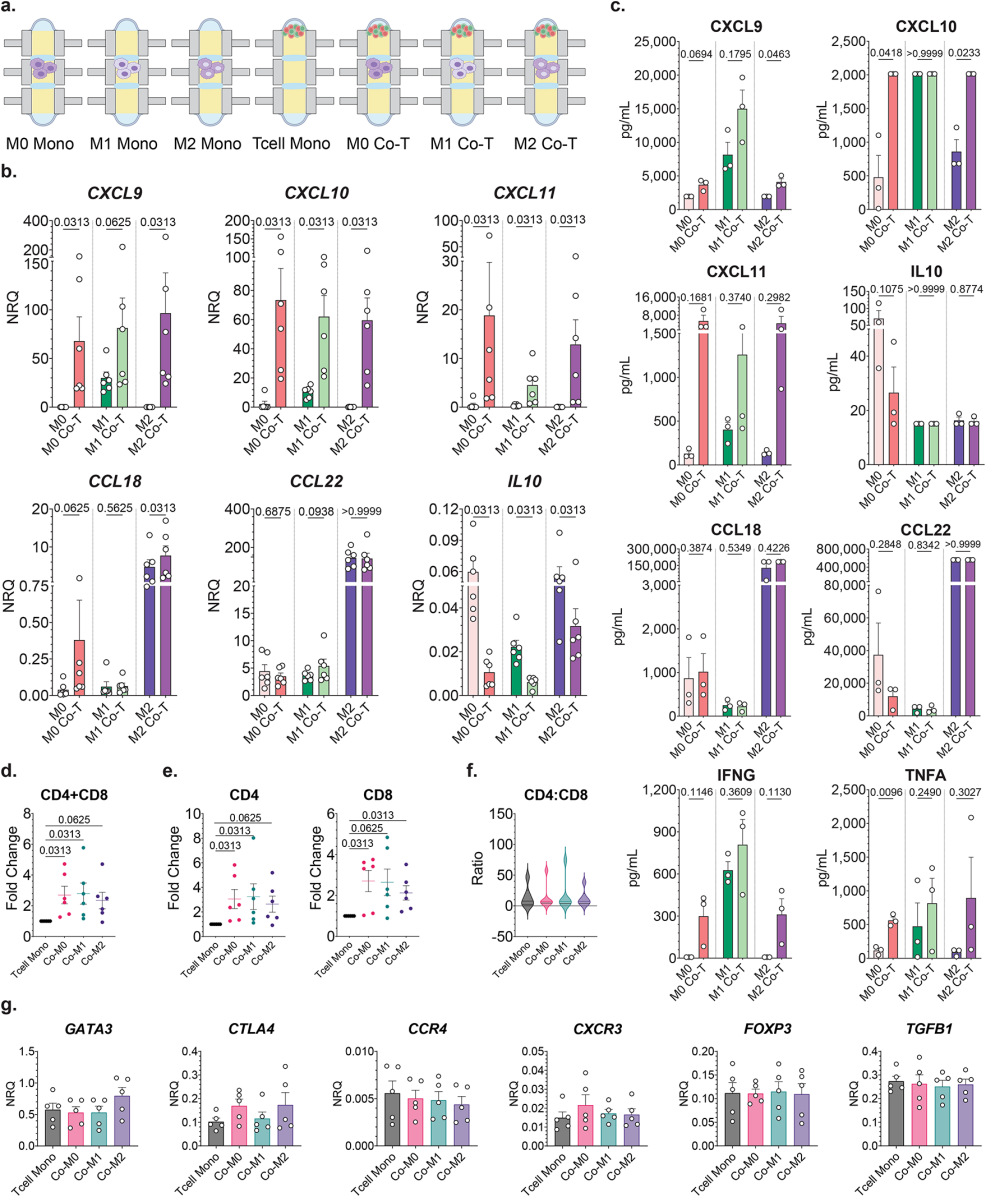
T cell impact on MDM polarization state. (**A**) Primary MDMs were cultured in mono-culture (M0) for 6 days and then polarized to M0, M1-like, or M2-like phenotypes for 3 days followed by either maintenance as mono-culture or combined into co-culture with autologous T cell cultures for the last 24 h. (**B**) RNA expression was interrogated for all polarized MDM mono-culture conditions and co-culture with T cells conditions. Data expressed as normalized relative quantity (NRQ), as related to house-keeping genes *RPLP0* and *POLR2A*. Paired Wilcoxon non-parametric test for corresponding polarization conditions; *n* = 6; error bar is mean with SEM. (**C**) Secreted cytokine and chemokine protein concentrations measured in cell culture supernatant media from polarized MDM mono-cultures and co-cultures with autologous T cells, expressed as pg/mL. Paired parametric test for corresponding polarization conditions; *n* = 3; Data represent mean ± SEM. (**D**–**F**) T cell migration analysis of T cell mono-culture and the three co-culture conditions was performed by in-chip confocal microscopy imaging. Data is expressed as each sample’s fold change in migration distance relative to its corresponding mono-culture condition, of (**D**) total T cell population and (**E**) CD4 or CD8 T cell sub-populations. One sample Wilcoxon signed-rank test with hypothetical value at 1 and ignoring values that match hypothetical value; *n* = 6; error bar is mean ± SEM. (**F**) Ratio of CD4 to CD8 absolute migration distances for T cell mono-culture and the MDM polarization co-culture conditions. One-way ANOVA nonparametric with Friedman post-hoc analysis comparing the mean rank of each column with the rank of every other column; *n* = 6; Data represent mean ± SEM. (**G**) T cell mRNA expression in T cell mono-culture and polarized MDM co-culture conditions. Data expressed as normalized relative quantity (NRQ) as related to house-keeping genes *RPLP0* and *POLR2*. One-way ANOVA nonparametric with Friedman post-hoc analysis comparing the mean rank of each column with the rank of every other column; *n* = 5; Data represent mean ± SEM.

To further evaluate the capacity of primary T cells to repolarize autologous immunosuppressive MDMs, we designed co-culture experiments that combined activated T cells with MDMs that were pre-polarized using established cytokine-based polarization protocols in Stacks^25,27^. Monocytes were differentiated in Stacks followed by polarization to M1-like phenotype or M2-like phenotype using IFNG, or IL4, respectively. Unpolarized M0 MDM cells were maintained with CSF1. The cells were either left in monoculture or co-cultured with autologous activated T cells for subsequent isolation and analysis (Fig. 6A).

First, we performed protein and mRNA expression analysis to validate the expected M1/M2/M0- like polarization profiles in our Stacks cultures (Fig. 6B,C). Consistent with the expected phenotypes, the M1-polarized MDMs had the highest expression of pro-inflammatory or M1-associated genes *CXCL9*,* CXCL10*,* CXCL11* and the M2-polarized MDMs expressed the highest levels of the anti-inflammatory or M2-associated genes including *CCL18* (C-C motif chemokine ligand 18), *CCL22* (C-C motif chemokine ligand 22) and *IL10*.

Next, we investigated the effect of autologous T cells on each of these three pre-polarized MDM groups. Despite the differences in the M1-, M2- and M0-like baseline profiles, when pre-polarized MDMs were co-cultured in the context of activated autologous activated T cells, all three upregulated pro-inflammatory *CXCL9*, *CXCL10*, and *CXCL11* gene expression a largely comparable level compared to matched mono-cultures, regardless of initial polarization state. Although the patterns of secreted protein analysis in MDM-T cell co-culture was largely consistent with the mRNA findings, not all changes in the protein analysis were statistically significant, likely due to interpatient variability and limited sample size, therefore further investigation of the secretome is necessary (Fig. 6C).

We also analyzed the anti-inflammatory/M2-associated gene expression in the MDMs following co-culture with the activated, autologous T cells. *CCL18* mRNA expression was significantly increased in the M2-like pre-polarized MDMs following co-culture with a similar trend in the M0 and M1-like pre-polarized cells (Fig. 6B,C). *IL10* mRNA expression decreased in all conditions following co-culture and there was no change in *CCL22*. In the supernatant, concentrations in both IL10 and CCL22 trended lower on the M0 condition following co-culture, however, there were no significant changes in the concentration of any of the M2-associated factors. Therefore, unlike the increase in pro-inflammatory gene expression with activated T cell co-culture, we did not find a consistent effect on anti-inflammatory/M2-associated genes. This pattern of changes in gene expression profiles was in accordance with the pattern that we saw in the mono, co-, and triculture models, where activated T cell co-culture had a more pronounced impact on pro-inflammatory gene expression than on anti-inflammatory gene expression signatures.

Next, we analyzed the effect of pre-polarized MDMs on T cell migration, and found that the presence of MDMs induced migration of CD4 and CD8 T cell subsets with similar trends regardless of pre-polarization states (Fig. 6D–F). Although T cell migration was induced in response to any MDM cells irrespective of polarization status, we observed a pattern that T cells co-cultured with M2-prepolarized macrophages migrated the least. Next, we investigated T cell mRNA polarization profiles in the context of pre-polarized MDMs but found no difference when MDMs were added to co-cultures, with the exception of a non-significant trend in GATA binding protein 3 (*GATA3*) upregulation in the presence of M2-like macrophage (Fig. 6G).

Collectively, our observations in these cytokine pre-polarization experiments supported our findings in the tri-culture models (tumor cell, MDM, T cell), which demonstrated that activated T cells had the potential to alter the polarization status of immunosuppressive MDMs to a more proinflammatory profile. While the impact of activated T cells on the MDM phenotype was somewhat dependent on prior polarization status, differences in that baseline gene expression profile did not appear to have a limiting impact on MDM-mediated induction of T cell migration or skewing towards proinflammatory phenotype in our TME model system.

## Discussion

**Fig. 7:**
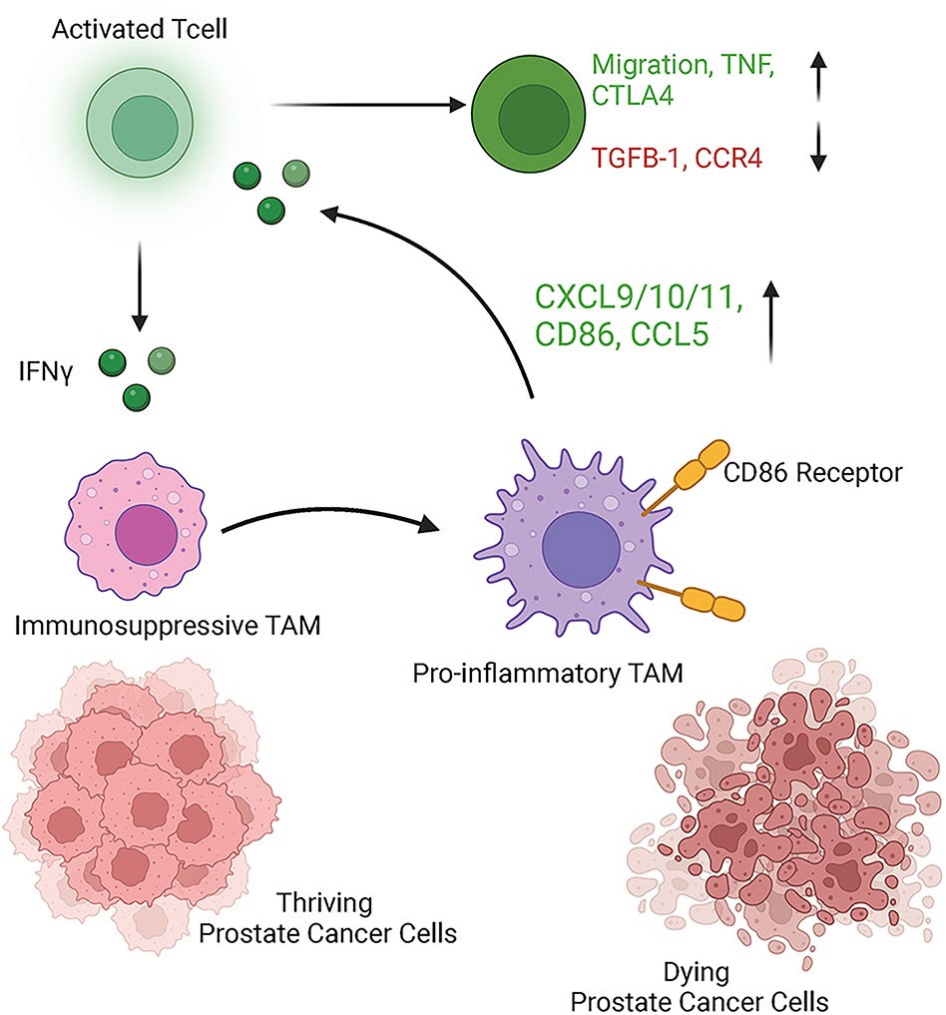
TAMs retain proinflammatory plasticity in the TME. Dynamic, reciprocal interaction between tumor-polarized macrophages and activated T cells allows paracrine reprogramming of TAM via IFNG secretion.

TAMs are a versatile cell population with the potential to either support or hinder tumor-directed immune responses^33^. This complexity as well as the limitations of traditional in vitro models, have presented major challenges in the comprehensive investigation of the role of TAMs in prostate cancer immune subversion. In this study, we leveraged novel microfluidic technologies and patient-derived cells to gain novel insight into the roles of TAMs in the prostate cancer immune microenvironment. These microscale technologies allowed us to perform multiplexed analysis of primary macrophages, autologous T cells, and tumor cells within the context of multicellular prostate tumor environments. Our data demonstrated that in the context of prostate tumor models or IL4 stimulation, primary MDMs expressed an immunosuppressive or M2-like phenotype. However, upon culture with activated T cells, these immunosuppressive MDMs remained inducible to upregulate expression of key proinflammatory cytokines and chemokines in response to T cell-derived IFNG (Fig. 7). Meanwhile, the MDM-derived proinflammatory factors, in turn, induced tumor-directed T cell migration and proinflammatory phenotype as part of a dynamic multilateral and multicellular interaction. In our model system, we simulated activated T cells with CD3/CD28 activation and cell to cell communication was limited to paracrine factors, therefore, future studies will need to investigate similar interactions in the context of tumor antigen specific T cells and cell-to-cell contact.

To our knowledge, our study provided the first direct evidence that primary T cells derived from men with prostate cancer could repolarize autologous macrophages to a proinflammatory phenotype in the context of both androgen dependent and independent prostate tumor models or cytokine-mediated in vitro M2 polarization. Furthermore, we also captured the first direct visualization of T cell migration and analysis of T cell phenotype in response to macrophage-mediated factors in the context of prostate tumor cells.

Our findings provide strong evidence that macrophages retain functional plasticity even in the context of immunosuppressive tumor networks and that primary immunosuppressive macrophages have the potential to actively respond to inflammatory T cell stimuli. These observations provide an explanation for the substantial, but sometimes conflicting data regarding the role of TAMs in the regulation of anti-tumor immune responses. In particular, prior studies have demonstrated that TAMs play a vital role in T cell exclusion from the TME while also playing an essential role in tumor-directed T cell response^18,34^. Our data suggest that these seemingly discordant roles are due to TAMs plasticity and their ability to respond to inflammatory stimuli within the TME. Therefore, while TAMs may engage in immunosuppressive activities within the context of certain TME conditions, an influx of activated T cells as a result of dynamic change in immune homeostasis in the TME may repolarize these TAMs to ultimately support tumor-directed T cell responses. These findings have particular significance with respect to strategic approaches that aim to target macrophages in order to enhance immunotherapeutic efficacy. Based on the data presented in this study, TAM-targeted therapies that aim to promote an anti-tumor immune response should focus on driving TAM reprogramming and not depletion or inhibition of recruitment, which are strategies that have been investigated^35^.

Further research will be critical to identify specific pathways blocking TAM repolarization within the TME and to develop therapeutic strategies that promote a proinflammatory TAM phenotype within prostate tumors. These studies will require incorporation of additional TME cell populations, such as cancer-associated fibroblasts (CAFs), endothelial cells, tumor epithelial cells, as well as other immune cells like MDSCs, and antigen-specific T cells to more faithfully reflect the prostate tumor infiltrate that collectively influence local immune homeostasis and tumor response. Additional investigation of how macrophages of differential polarization states including tumor-naive M0, M1 or M2-like MDMs and tumor pre-polarized TAMs alone effect autologous T cell behaviors may shed light of other potentially targetable mechanisms to disrupt immune suppressive TME interactions.

Spatial exclusion of T cells has been previously captured in prostate tumor rendering these foci a cold immune microenvironment and hinder anti-tumor response. TME elements including TAMs and CAFs have been proposed to contribute to mechanisms behind T cell exclusion. Future studies in our multi-cellular TME model system incorporating variable spatial reconfigurations will explore how these TME cell populations drive T cell exclusion and whether the pathways that mediate this effect on T cells can be targeted. Additionally, immune check-point therapies should be assessed in combination with other therapies, including hormone therapy, radiation or chemotherapy to facilitate TAM reprogramming and restore T cell infiltration.

Integrated microfluidic cell culture platforms, such as Stacks, have the potential to significantly contribute to this research by enabling spatio-temporal investigation of complex and dynamic multi-cellular niches, such as the prostate TME and can advance our understanding of these networks to expedite discovery and allow rapid assessment of novel therapies that induce proinflammatory TAM phenotypes. Such therapies could then be combined with other immunotherapy strategies, including positive or negative immune checkpoint therapies and epigenetic modifying agents, to improve efficacy in patients with prostate cancer as well as potentially enhance the utility of immune therapeutics in other malignancies.

## Methods

### Human tissue collection and PBMC isolation

Blood samples were collected from 25 patient donors with prostate cancer after receiving written informed consent under a protocol approved by the Institutional Review Boards at the University of Wisconsin-Madison (#2014-1214) and at the William S. Middleton Memorial Veteran’s Hospital, Madison, WI, USA (#WI-018). Research has been performed in accordance with the Declaration of Helsinki. Blood specimens were collected in vacutainer tubes (BD Biosciences, Franklin Lake, NJ, USA) with EDTA anticoagulant. Whole blood was diluted 1:1 with Hank’s balanced salt solution (HBSS, Lonza Group, Basel, Switzerland) before being underlaid with 10 ml of Ficoll-Paque PLUS (GE Healthcare, Cat# 45-001-750) for gradient centrifugation. CD14^+^ monocytes were enriched from PBMCs using LS MACS columns following incubation with anti-CD14 magnetic beads (Miltenyi Biotec Inc., Bergisch Gladbach, North Rhine-Westphalia, Germany #130-050-201). T cells were subsequently isolated from the CD14^−^ cell fraction on LS columns using the Pan T cell isolation kit (Miltenyi Biotec inc., Germany # 130-096-535).

### Cell Culture

LNCaP (ATCC, Cat# CRL-1740, RRID: CVCL_0395), 22Rv1 (ATCC Cat# CRL-2505, RRID: CVCL 1045), patient-derived primary T cells, and autologous macrophage were cultured in Stacks by seeding as a monolayer on a collagen-fibronectin matrix, consisting of 79% collagen I (Advanced BioMatrix, Carlsbad, CA USA #5005), 1.5% fibronectin (Sigma-Aldrich, Millipore Sigma, Burlington, MA, USA #F1141) prepared according to manufacturer guidelines. Moisture was retained by storing Stacks inside of a humidifying chamber. LNCaP and 22Rv1 cells were acquired from ATCC and were cultured in RPMI1640 media with L-Glutamine (Corning Life Sciences, Tewksbury, MA, USA # 10-040-CV), 10% FBS (Gibco, Thermo Fisher Scientific, Waltham, MA USA #10-438-026) and 2% penicillin/streptomycin (Hyclone, Cytivia, Marlborough, MA USA # SV30010). Single cell suspensions of LNCaP and 22Rv1 cells were harvested at log phase and were seeded onto Stacks hydrogel wells at a concentration of 1 × 10^6^ or 3 × 10^5^ cells per ml, respectively. Patient-derived monocytes and T-cells were cultured in RPMI1640 media with L-Glutamine, 10% FBS, 5% Glutamax (Gibco, Thermo Fisher Scientific, Waltham, MA USA #35050061), and 2% penicillin/streptomycin. Using established protocols, CD14^+^ monocytes were differentiated into macrophages using 50 ng ml^− 1^ colony stimulating factor 1 (CSF1) (Tonbo Biosciences, Cytek, San Diego, CA USA #21-8789-U010) with an initial seeding concentration of 3 × 10^6^ cells per ml. On Day 4, 50 ng ml^− 1^ IFNG (Tonbo Reagents, Cytek Biosciences, San Diego, CA USA #21-8319-U020) or 40 ng ml^− 1^ interleukin 4 (IL4) (Tonbo Reagents, Cytek Biosciences, San Diego, CA USA #21-8044-U005) were added for an additional 3 days to induce M1 and M2 polarization, respectively. To maintain unpolarized/M0 phenotype, 50 ng/mL CSF1 was readministered at Day 4 for an additional 3 days. T cells were activated with Dynabeads, Invitrogen, Human T-activator CD3/CD28 beads (Thermo Fisher Scientific, Waltham, MA, USA #11131D) and were maintained in suspension in 24-well plates with an initial concentration of 5 × 10^5^ cells in 0.5 ml. For migration experiments, T cells were seeded on Stacks hydrogel wells at a concentration of either 5 × 10^5^ or 2.5 × 10^4^ cells per ml and allowed to migrate for 24 h. For IFNG neutralization experiments, either 1 µg ml^− 1^ anti-IFNG neutralizing antibody (R&D Systems by bio-techne, Minneapolis, MN USA Cat# AF-285-NA, RRID: AB_354445) (1:1000), 1 µg ml^− 1^ IgG control R&D Systems by bio-techne, Minneapolis, MN USA Cat# AB-108-C, RRID: AB_354267) (1:1000), or 0.1% 1xPBS (1:1000) were added to a T cell resuspension media immediately before the addition of the T cells to their culture layer for the 24 h migration period.

## Stacks devices

Prior to culture, Stacks plates (Protolabs, Maple Plain, MN USA #1121-5161-007) were prepared by sonication in 100% isopropanol for 60 min and washed in deionized water^24^. Stacks devices, 3D holders, Nunc OmniTray Single-Well Plate (Thermo Fisher Scientific, Waltham, MA USA #140156), and not tissue culture-treated 245 mm Square BioAssay Dish with Handles ( Corning Life Sciences, Tewksbury, MA USA #431111) were sterilized by 20 min UV light treatment on each side. Cells were plated in the Stacks device and then allowed to adhere or migrate through the matrix as applicable. When stacking 2 or more devices, media was removed from the top and bottom leaving only a small volume of residual media to prevent gas bubble formation during stacking. Stack devices were placed in a three-layer humidifying chamber including a sterile sponge soaked in sterile ddH_2_O in a Nunc OmniTray.

## Nucleic acid extraction and quantitative RT-PCR

Cells were removed from hydrogel matrix via application of 5 µl of 1 mg ml^− 1^ collagenase I (Sigma-Aldrich, Millipore Sigma, Burlington, MA, USA #C9697) for matrix digestion prior to transferring single cell suspensions to 96-well plates for subsequent lysis containing 40 µl of lysis/binding buffer solution + 10 µl of LB washed beads per well. Stacks culture wells were washed with an additional 10 µl of lysis/binding buffer to maximize cell recovery from Stacks microwells. mRNA isolation was performed using Invitrogen Dynabeads mRNA DIRECT Micro Purification Kit (Thermo Fisher Scientific, Waltham, MA, USA #61012). The lysate was washed with 100 µl Buffer A x 2 and 100 µl Buffer B x 1, then suspended in 16 µL of Nuclease-Free Water (Promega, Madison, WI USA # P1197).

The mRNA elution sample was reverse transcribed using 4 µl Superscript IV VILO Master Mix (Invitrogen, Thermo Fisher Scientific, Waltham, MA, USA #11756050), according to manufacturer’s directions using C1000 Touch Thermal Cycler (Bio-Rad Laboratories, Hercules, CA, USA). The subsequent cDNA solution (6.3 µl) was then amplified using 12.5 µl TaqMan PreAmp Master Mix (Applied Biosystems, Thermo Fisher Scientific, Waltham, MA, USA #4488593) plus 6.25 µl of a primer mix. The primer mix contained 10 µl of each available primer and TE buffer to bring the mix up to 1000 µl final volume. 10 cycles of pre-amplification were performed according to manufacturer’s directions and the final reaction volume was then diluted by adding 200 µl nuclease-free water (Promega, Madison, WI, USA # P1197). For TaqMan qPCR assays, 2.5 µl of diluted cDNA template was mixed with 5 µl TaqMan Fast Advanced Master Mix for qPCR (Applied Biosystems, Thermo Fisher Scientific, Waltham, MA, USA #4444557), 0.5 µl TaqMan Gene Expression Assay *CCL18* (Hs00268113_m1), *CCL22* (Hs01574247_m1), *CXCL9* (HS00171065_m1), *CXCL10* (Hs01124252_g1), *CXCL11* (Hs04187682_g1), *IL10* (Hs00961622_m1), *CXCR3* (Hs00171041_m1), *CCR4* (Hs01396342_m1), *CTLA4* (Hs00175480_m1), *FOXP3* (Hs01085834_m1), GATA binding protein 3 (*GATA3*) (Hs00231122_m1), *TGFB1* (Hs00998133_m1), *CCL2* (Hs00234140_m1), and *CCL5* (HS00982282_m1); and housekeeping genes: ribosomal protein lateral stalk subunit P0 (*RPLP0*) (Hs00420895_gH) and RNA polymerase II subunit A (*POLR2A*) (Hs00172187_m1) *(*Life Technologies, USA) and 2 µl nuclease-free water (Promega, Madison, WI, USA # P1197). Each reaction was amplified in duplicate using a QuantStudio 5 Real-Time PCR Instrument (Applied Biosystems, Life Technologies, RRID: SCR_020240) via the Comparative CT Fast protocol available on the machine. Analysis was performed using the Thermo Fisher Connect online application (ThermoFisher Connect Platform, RRID: SCR_023441).

### Confocal microscopy analysis

For confocal microscopy analysis, cells were then fixed at their location within the collagen-fibronectin matrix (eBioscience, Thermo Fisher Scientific, Waltham, MA, USA; # 00-5521-00), washed, and then stored at 4 °C. For T cell migration analysis, staining was performed via overnight incubation at 4 °C of a 1x PBS + 10% FBS solution comprised of 20 mM Hoechst 33342 (1:250), anti-CD4 Brilliant Blue 515 (BD Biosciences, Franklin Lakes, NJ USA #564419) (1:50), and anti-CD8 subunit alpha (CD8) (BD Biosciences, Franklin Lakes, NJ USA #555367) (1:100). Each well was imaged in its entirety on a Yokogawa CSU-W1 Spinning Disk Confocal Microscope (Nikon, Tokyo, Japan) at 10x magnification with 10 μm z-layers. Images were analyzed with NIS-Elements software version 5.20.02 (RRID: SCR_014329; Nikon Instruments, Melville, NY, USA). Individual cells were identified using spot detection function in the Hoechst channel to identify each nucleus. CD4^+^ or CD8^+^ populations were identified by thresholding for signal intensities in the 488 nm and 561 nm channels, respectively. The number of migrated cells was determined by quantifying the number of CD4^+^ and CD8^+^ cells in all z-layers beyond 400 μm from the top of the well. The 400 μm threshold was selected because this distance corresponded to the average depth of curvature of the hydrogel surface. For macrophage analysis, staining was performed via overnight incubation at 4 °C of a 1x PBS + 10% FBS solution comprised of 20mM Hoechst (1:250), anti-CD86 molecule (CD86) (BD Biosciences, Franklin Lakes, NJ USA Cat#564544, RRID: AB_2744453) (1:50), anti-CD163 molecule (CD163) (BioLegend, San Diego, CA USA Cat#326507, RRID: AB_893268) (1:50), and anti-CD14 molecule (CD14) (BioLegend, San Diego, CA USA Cat#301850, RRID: AB_2564138) (1:50) antibodies. Images were analyzed with NIS-Elements software version 5.20.02 (RRID: SCR_014329; Nikon Instruments, Melville, NY, USA). To account for differences in signal intensity in each z-layer, the intensity ratio was calculated by dividing the mean fluorescent intensity of the CD86 signal by the mean fluorescent intensity of the Hoechst stain fluorescence for each condition replicate.

### Supernatant analysis

Supernatant media was collected from Stacks microwells following the designated culture period. Media from multiple wells of the same culture condition were combined to a volume of 20 µl and diluted in 40 µl of media prior to centrifugation at 300 g for three minutes. 53 µl of the supernatant was removed, snap frozen on dry ice, and stored at -80 °C until analysis. Samples were analyzed using a custom multiplexed Quantikine kit according to the manufacturer’s protocol (R&D Systems, Minneapolis, MN, USA) using a MAGPIX System (Luminex LTC, DiaSorin, Saluggia, Italy). Samples, standards, and blanks were assayed in duplicate. Cytokine concentration was quantified using the standard curve generated by the 4-PL curve fit analysis tool in GraphPad Prism version 9.5.1.1 (RRID: SCR_002798). Data values were limited to a minimum value equal to the sixth standard’s theoretical concentration and to a maximum value equal to the concentration interpolated for a mean fluorescent intensity (MFI) value up to 1.5x of the MFI range for the standards. The minimum value of the data range for Fig. 4 was extended out to the ninth standard’s theoretical value due to the material used in this assay having been diluted an additional 2x with calibrator dilutant from the assay kit, for a total dilution of 6x.

### Statistical analysis

All statistical tests were performed using GraphPad Prism version 9.5.1.1. One-way analysis of variance (ANOVA) nonparametric with post hoc Friedman’s correction comparing the mean rank of each condition with the mean rank of every other condition, ordinary one-way analysis of variance ANOVA with post hoc comparison of the mean rank of each condition with the mean rank of every other condition, one-sample Wilcoxon signed-rank test, t-test paired nonparametric, t-test paired parametric, and one-way ANOVA nonparametric with post hoc Friedman’s correction comparing specific conditions of focus, were run and indicated as such in figure legends.

## Electronic supplementary material

Below is the link to the electronic supplementary material.

Supplementary Material 1

Supplementary Material 2

## Data Availability

Data is provided in supplementary information files.
