# Supplementary material for: Dynamic reciprocal interactions between activated T cells and tumor associated macrophages drive macrophage reprogramming and proinflammatory T cell migration within prostate tumor models
Source: Sci Rep. 2024 Oct 16;14:24230. doi: 10.1038/s41598-024-75265-9 (PMC11484957; doi:10.1038/s41598-024-75265-9)
Supplement: Supplementary file 1 — Supplementary Material 1 [file 41598_2024_75265_MOESM1_ESM.pdf]

SUPPLEMENTARY MATERIAL

Dynamic reciprocal interactions between activated T cells and tumor associated macrophages drive macrophage reprogramming and proinflammatory T cell migration within prostate tumor models

Erika Heninger, Matthew Thomas Breneman, Emma Elizabeth Recchia, Sheena Catherine Kerr, Reyna Elvan Dogru, Marina Nasrin Sharifi, Aaron Matthew LeBeau, and David Kosoff,

Supplementary Table S1. Patient Characteristics

	Number of Patients
Total Number of Donors	25
Patients with Locally Advanced Prostate Cancer	4
Patients with Metastatic Prostate Cancer	21
Patients with Castrate Sensitive Prostate Cancer	15
Patients with Castrate Resistant Prostate Cancer	10

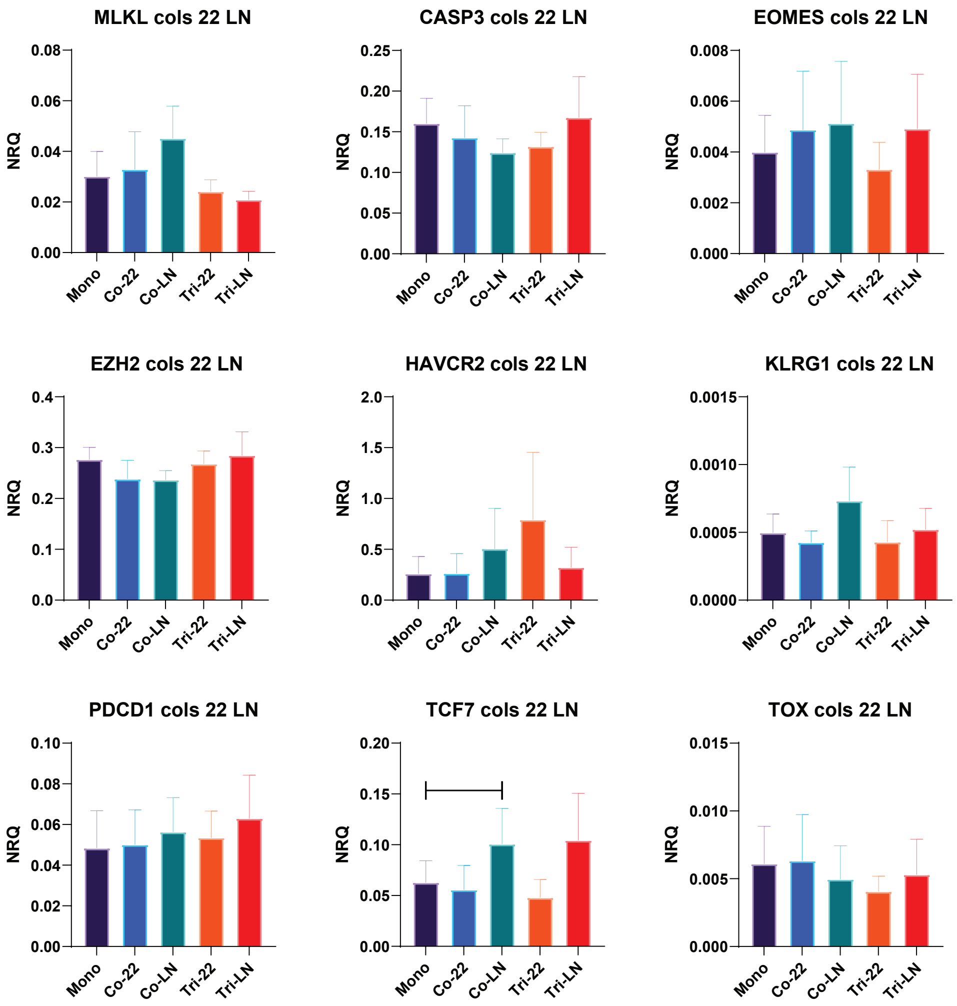

Supplementary Figure 1. Supplemental tri-culture mRNA expression data. mRNA expression in T cells was interrogated in T cell mono-, tumor co-culture, and tumor-MDM tri-culture conditions. Data expressed as normalized relative quantity (NRQ), as related to housekeeping genes *RPLP0* and *POLR2*; n=5.

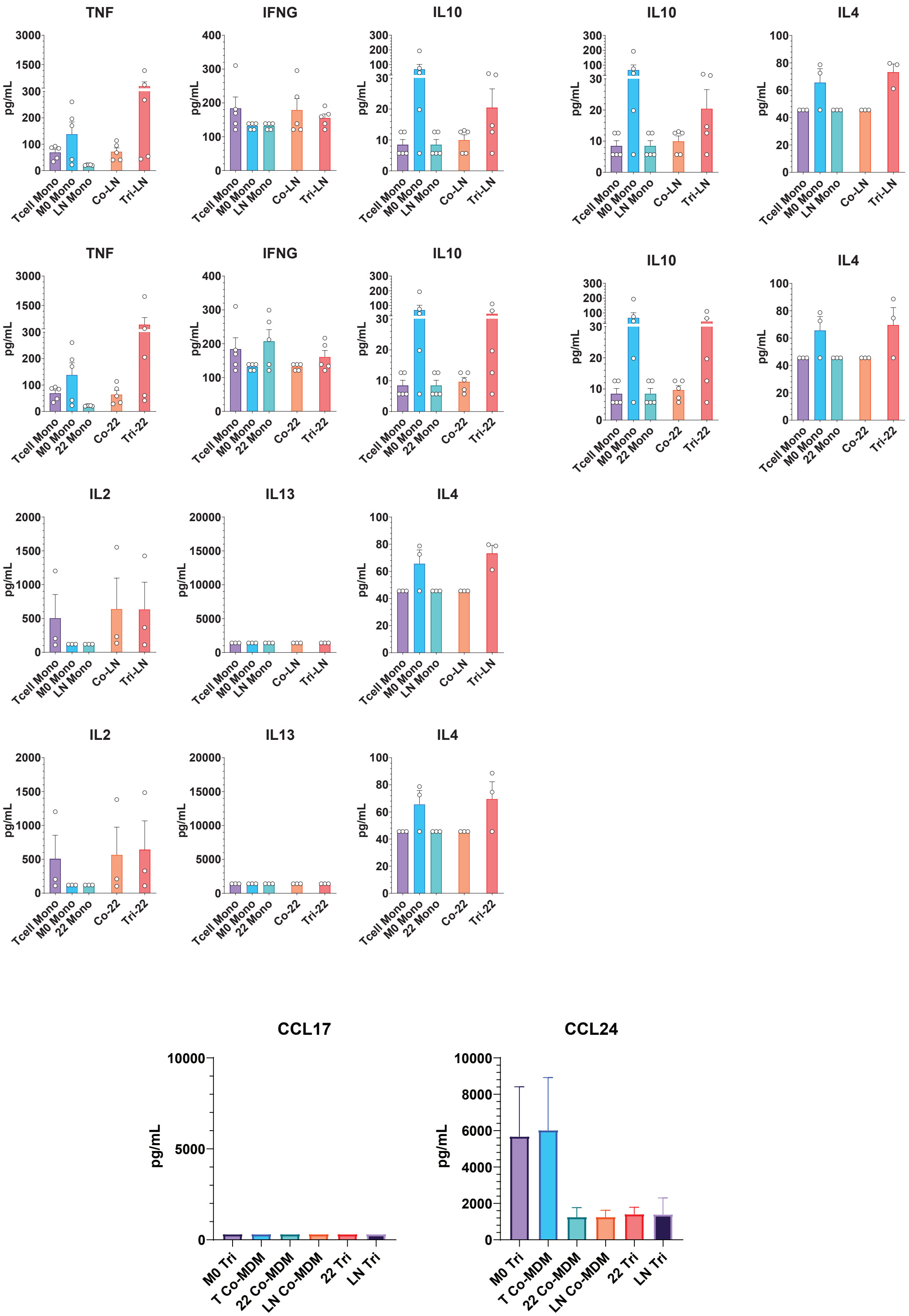

Supplementary Figure 2. Supplemental tri-culture secreted protein analysis. Supernatant was collected from five unique cellular conditions and analyzed for cytokine and chemokine expression levels using a multi-analyte bead assay. Data expressed as concentration (pg/mL); n=5.
